# Intradural lumbar disc herniation of L2–L3: A case report and literature review

**DOI:** 10.3389/fsurg.2022.1047974

**Published:** 2023-01-13

**Authors:** Xipeng Chen, Yuanpei Cheng, Han Wu

**Affiliations:** Department of Orthopedics, China-Japan Union Hospital of Jilin University, Changchun, China

**Keywords:** lumbar disc herniation, intradural lumbar disc herniation, lower back pain, lower extremity weakness, case report

## Abstract

**Background:**

Intradural lumbar disc herniation (ILDH), especially upper lumbar intradural disc herniation, is a rare type of lumbar disc herniation (LDH). However, it may have severe and complex symptoms, causing serious impact on the patients. Additionally, it is difficult to be diagnosed with limited experience. Few studies on L2–L3 ILDH have been reported in the literature. This study presents such a case and reviews the incidence, etiology, symptoms, diagnosis and treatment of this disease, so as to provide guidance and experience for clinicians.

**Case presentation:**

A 27-year-old male patient had a one-month history of severe lower back pain and left lower extremity weakness after lumbar sprain. He could not walk due to progressive symptoms. Physical examination revealed that straight leg raising and femoral nerve stretch tests on the left side were positive. Magnetic resonance imaging of lumbar showed an intradural disc protruding into the ventral dural sac at the L2–L3 level. He was diagnosed ILDH of L2–L3, finally. An urgent operation was performed to remove the intradural disc fragment. The patient's symptoms improved significantly, postoperatively. After eight months of follow-up, he returned to normal life with only slight lower back pain.

**Conclusions:**

ILDH at the L2–L3 level is an extremely rare type of LDH. Its diagnosis often requires a combination of symptom, physical examination, and imaging examination due to no typical symptoms or imaging features. A detailed preoperative plan including the definition of the position, calcification, migration, and adhesion of intradural intervertebral discs to decrease the risk of surgery, prevent the occurrence of complications, and promote postoperative prognosis of patients.

## Introduction

Intradural lumbar disc herniation (ILDH) is a rare type of lumbar disc herniation (LDH), and its incidence is 0.04–0.33% of LDH (1). ILDH occurs more rarely in the upper lumbar spine than the lower lumbar spine (1). The etiology of ILDH is still uncertain. The most widely accepted mechanism is adhesion between the posterior longitudinal ligament (PLL) and ventral dural sac. For ILDH, there are atypical and complex symptoms, such as low back pain, radiation pain, or lower extremity weakness. More seriously, ILDH may cause cauda equina syndrome (CES) (2). Prompt surgery is used in the treatment of ILDH, so as to alleviate severe symptoms, avoid aggravation of the condition, and prevent adverse events. ILDH may be misunderstood as intradural extramedullary tumor, hematomas, or synovial cyst based on preoperative radiographic imaging (3, 4). It is difficult to make definitive preoperative diagnosis, which increases the risk of surgery and reduces the prognosis. According to literature reports, there have been few studies on L2–L3 ILDH. The purpose of this study is to describe a case with L2–L3 ILDH, to review the incidence, etiology, symptoms, diagnosis and treatment of this disease, and to provide guidance and experience for orthopedic surgeons.

## Case presentation

**Figure 1:**
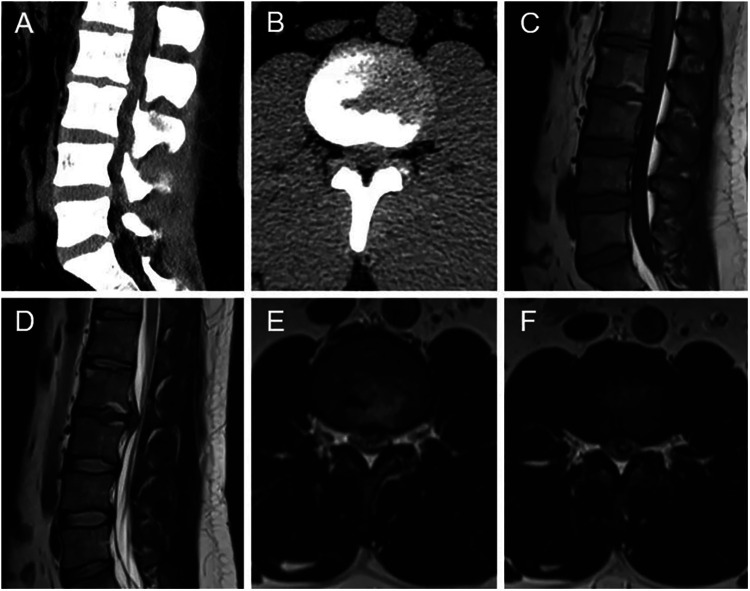
Preoperative radiographic images (**A–F**). (**A,B**) CT showed intervertebral disc herniation, loss of disc height, and osteophyte hyperplasia at the L2–L3 level. Sagittal T1-weighted (**C**) and T2-weighted (**D**) MRI revealed extruded intervertebral disc, and loss of continuity of the PLL at the L2–L3 level. (**E,F**) Axial T2-weighted MRI demonstrated the intradural mass.

**Figure 2:**
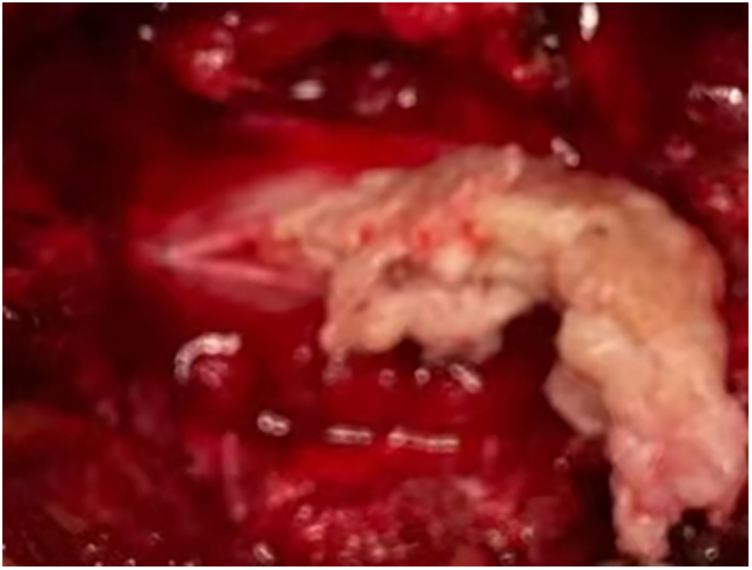
Interoperative image revealed the extruded intervertebral disc.

**Figure 3:**
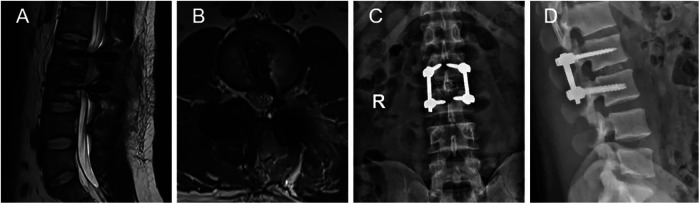
Postoperative radiographic images (**A–D**). (**A,B**) MRI revealed that the extruded lumbar intervertebral disc was removed. (**C,D**) Plain radiographs showed interbody fusion with posterior instrumentation.

A 27-year-old male patient who suffered from severe lower back pain and left lower extremity pain due to lumbar sprain a month ago. The patient accepted some conservative treatments such as non-steroidal anti-inflammatory drugs and bed rest. However, the symptoms did not alleviate, or even worsened. He experienced the left lower extremity weakness, and then he could not walk due to progressive symptoms. No further treatment was adopted. The movement was limited, and the patient failed to take part in social activities or even look after himself. Therefore, the patient was admitted to our hospital for treatment. He denied having a similar medical history and a family history of genetic disorders. Physical examination revealed muscle weakness in the left lower extremity, especially left ankle dorsiflexion and great toe dorsiflexion. The left-side straight leg raising and femoral nerve stretch tests were positive. The bowel and bladder functions were normal. Lumbar computed tomography (CT) showed that intervertebral disc herniation, loss of disc height, and osteophyte hyperplasia at the L2–L3 level (Figures 1A,B). Lumbar magnetic resonance imaging (MRI) demonstrated that extruded intervertebral disc, loss of continuity of the PLL, and intradural masses at the L2–L3 level (Figures 1C–F). The masses with T1-weighted and T2-weighted signals equaled to those of intervertebral discs. ILDH was preoperative diagnosis on the basis of medical history, symptoms, physical examination, and radiographic findings. Its differential diagnosis, such as intradural tumor and cyst, still needs to be considered. A laminectomy was performed under general anesthesia. The dura sac in the spinal canal was exposed by removing the ligamentum flavum. Extruded intervertebral disc was not found in the extradural space, but there were tight adhesions between the PLL and the ventral dural sac. A durotomy of the dorsal dural sac was completed and the nerve rootlets were separated. There was a mass revealed disc-like substance in the dura sac (Figure 2). A defect of the ventral dural sac was observed after the mass was removed. The dorsal and ventral dural sacs were sutured continuously. Cerebrospinal fluid (CSF) leakage was not discovered, and there was no obvious compression of the nerve root and dural sac after careful inspection. Transforaminal lumbar interbody fusion and transpedicular screw fixation were performed to prevent the recurrence of ILDH and spinal instability. Subsequently, the incision was sutured and the drainage was placed. Antibiotics were injected intravenously for 3 days after the surgery. Postoperative radiographs revealed that the extruded intervertebral disc was removed and the position of internal fixation was good (Figure 3). The patient's pain improved significantly, postoperatively. The patient was followed up by outpatient review. Lower extremity weakness gradually improved, and no complications such as infection, cerebrospinal fluid leakage, or epidural hematoma were found during the follow-up period. Eight months postoperatively, the patient could return to normal life, walk freely, and participate in social activities with only slight lower back pain. Physical examination showed the muscle strength of the left lower extremity was significantly improved compared with that before surgery. The left-side straight leg raising and femoral nerve stretch tests were negative. The visual analogue scale score and the Oswestry disability index significantly improved from 7 and 72 preoperatively to 2 and 18, respectively. The patient was satisfied with the efficacy.

## Discussion

ILDH was first described by Dandy in 1942 (5). ILDH is rare with an incidence of 0.04–0.33% of LDH, however, ILDH at the L2–L3 level is even rarer with an incidence of only 9.5% of all ILDH (1). To our knowledge, there are 21 patients with ILDH at L2–L3 level in the literature (Table 1). ILDH often occurs in patients over 50 years old, and it is more common in men than in women (1). We reported a male patient with ILDH at the L2–L3 level who was only 27 years old.

**Table 1:** Summary of reported cases of ILDH at the L2–L3 level.

Study	Year Published	Age	Sex	Symptoms	Surgical method	Outcome
Cantini (6)	1986	72	Female	LBP, LLP, CES	Not clear	Not clear
Lee (7)	2020	32	Male	LLP, LLW	Laminectomy	Improvement
Crivelli (8)	2017	73	Male	LLP	Not clear	Not clear
Hidalgo-Ovejero (9)	2004	64	Female	LBP, CES	Laminectomy and internal fixation	Improvement
Sasaji (10)	2011	56	Male	LLP, LLW	Laminectomy	Improvement
Moon (11)	2020	65	Male	LLP	Microscopic discectomy	Improvement
Kim (12)	2018	78	Male	LBP	PELD	Improvement
Karabekir (13)	2006	46	Male	LBP, LLP	Interlaminar fenestration	Improvement
Koc (14)	2001	59	Male	LBP, LLP	Laminectomy	Improvement
Kobayashi (15)	2014	67	Female	LLP	Interlaminar fenestration	Improvement
D'Andrea (16)	2004	72	Female	LBP, LLP	Hemilaminectomy	Improvement
Han (17)	2009	40	Male	CES	Subtotal laminectomy	Improvement
Kataoka (1)	1989	33	Male	LBP, LLP	Laminectomy	Improvement
Daffner (18)	2015	68	Female	LBP, LLP, LLW	Laminectomy	Improvement
Whittaker (19)	1994	66	Male	LLP	Not clear	Not clear
Hida (20)	1999	60	Female	LBP, LLW	Laminectomy	Improvement
Sakai (21)	2007	72	Female	LLP	Laminoplasty	Improvement
Kim (22)	2012	54	Male	LBP, LLP	Laminectomy	Improvement
Tempel (23)	2016	56	Male	CES, LLW	Laminectomy	Improvement
Chen (24)	2021	63	Female	LBP, LLP	Laminectomy and internal fixation	Improvement
Pholprajug (25)	2022	67	Male	CES, LLW	TLIF	Improvement

LBP, low back pain; LLP, lower extremity pain; LLW, lower extremity weakness; CES, cauda equina syndrome; PELD, percutaneous endoscopic lumbar discectomy; TLIF, transforaminal lumbar interbody fusion.

The etiology of ILDH remains uncertain. Adhesions of the PLL and the ventral dural sac may be a predisposing factor for ILDH and could occur in congenital conditions such as congenital lumbar spinal stenosis, or in chronic degenerative diseases (17, 26–28). The adhesion may cause dural erosion, followed by herniation of the intervertebral disc into the dural sac. D'Andrea et al. (16) found that 33.3% of ILDH patients had a history of previous surgery. Previous surgery may contribute to intradural disc herniation due to postoperative adhesions of the PLL and the ventral dural sac. Jang et al. (29) revealed that an acute trauma may lead to the occurrence of ILDH. The symptoms of ILDH are atypical and complex, including low back pain, radiation pain, or lower extremity weakness, which is more serious than epidural disc herniation. Moreover, the incidence of CES in ILDH is higher than extradural lumbar disc herniation (2).

The diagnosis of ILDH is still difficult and challenging. Some characteristics of imaging should be noticed to rule out some differential diagnoses. Hidalgo-Ovejero et al. (9) demonstrated that an intradural herniated disc was associated with the presence of epidural gas on CT scans and suggested that ILDH should be considered when epidural gas was observed. Sasaji et al. (10) reported that the “Y sign” formed by the ventral dura and arachnoid on sagittal T2-weighted MRI could contribute to the diagnosis of ILDH. Choi et al. (30) found that the “hawk-beak sign” on the axial imaging and loss of continuity of the PLL on T2-weighted sagittal MRI could indicate the occurrence of ILDH. Wasserstrom et al. (31) reported that ILDH was diagnosed by rim enhancement of herniated disc on gadolinium-enhanced MRI. Rim enhancement on gadolinium-enhanced MRI were used to rule out some differential diagnoses such as meningioma and schwannoma (32, 33). Note that rim enhancement of herniated disc occurred due to peripheral neovascularization and chronic granulation tissue (33). Myelography could be used to diagnose ILDH. Kataoka et al. (1) revealed that 71% and 15% of ILDH patients showed complete block and incomplete block in myelogram, respectively. However, myelography failed to rule out intradural tumors and huge central herniation (34, 35).

The optimal and effective treatment of ILDH is surgery that completely removes intradural intervertebral discs. Laminectomy is considered as the mainstream procedure and is widely used to treat ILDH. CSF leakage may be a serious complication. Pedaballe et al. (36) concluded that the defect of the ventral dura should be sutured. Some hemostatic materials should be placed on the ventral dural sac to prevent the occurrence of CSF leakage (37). A study by Serikyaku et al. (38) demonstrated that no postoperative CSF leakage was found in all patients of ventral durotomy without repair. The treatment of ventral dural defect remains controversial. Note that intradural migration of intradural disc fragment was easily misdiagnosed as intradural extramedullary tumor, and also increased the risk of surgical failure (22). Kobayashi et al. (15) reported that transforaminal lumbar interbody fusion was performed to treat ILDH, so as to prevent recurrence. Recently, Kim et al. (12) has been found that percutaneous endoscopic lumbar discectomy was successfully conducted in the treatment of ILDH. However, Moon et al. (11) reported the conversion to microscopic surgery after the failure of percutaneous endoscopic lumbar discectomy for treatment of ILDH.

There are some limitations to our study. First, pathological examination of extruded intervertebral disc was not performed. Second, the follow-up period of the patient was short. Third, only one case was analyzed in this study.

## Conclusions

ILDH at the L2–L3 level is an extremely rare type of LDH. The etiology of ILDH is multifaceted. Its symptoms and preoperative radiological findings are often atypical. The diagnosis of ILDH is often based on the combination of symptom, physical examination, and imaging examination, and is confirmed during operation. Orthopedic surgeons should make detailed preoperative plans including the definition of the position, calcification, migration, and adhesion of intradural intervertebral discs to decrease the risk of surgery, prevent the occurrence of complications, and promote postoperative prognosis of patients.

## Data Availability

The raw data supporting the conclusions of this article will be made available by the authors, without undue reservation.
